# Addressing IGHV Gene Structural Diversity Enhances Immunoglobulin Repertoire Analysis: Lessons From Rhesus Macaque

**DOI:** 10.3389/fimmu.2022.818440

**Published:** 2022-03-28

**Authors:** Mateusz Kaduk, Martin Corcoran, Gunilla B. Karlsson Hedestam

**Affiliations:** Department of Microbiology, Tumor and Cell Biology, Karolinska Institutet, Stockholm, Sweden

**Keywords:** antibody repertoire, next generation sequencing, macaques, immunoglobulin germline genes, structural variation, databases

## Abstract

The accurate germline gene assignment and assessment of somatic hypermutation in antibodies induced by immunization or infection are important in immunological studies. Here, we illustrate issues specific to the construction of comprehensive immunoglobulin (IG) germline gene reference databases for outbred animal species using rhesus macaques, a frequently used non-human primate model, as a model test case. We demonstrate that the genotypic variation found in macaque germline inference studies is reflected in similar levels of gene diversity in genomic assemblies. We show that the high frequency of IG heavy chain V (IGHV) region structural and gene copy number variation between subjects means that individual animals lack genes that are present in other animals. Therefore, gene databases compiled from a single or too few animals will inevitably result in inaccurate gene assignment and erroneous SHM level assessment for those genes it lacks. We demonstrate this by assigning a test macaque IgG library to the KIMDB, a database compiled of germline IGHV sequences from 27 rhesus macaques, and, alternatively, to the IMGT rhesus macaque database, based on IGHV genes inferred primarily from the genomic sequence of the rheMac10 reference assembly, supplemented with 10 genes from the Mmul_051212 assembly. We found that the use of a gene-restricted database led to overestimations of SHM by up to 5% due to misassignments. The principles described in the current study provide a model for the creation of comprehensive immunoglobulin reference databases from outbred species to ensure accurate gene assignment, lineage tracing and SHM calculations.

## Introduction

Non-human primates are frequently used for studies of vaccine- and infection-induced B cell responses with rhesus macaques (*Macaca mulatta*) being the most common model. Central for studies of B cell function is the isolation of antigen-specific monoclonal antibodies (mAbs), which facilitates detailed biochemical, structural, and functional analyses of the response (1–8). The availability of mAbs also allows studies of Ab affinity maturation and Ab clonal relationships, requiring examination of Ab sequences at the genetic level. If combined with bulk B cell receptor repertoire sequencing (Rep-seq), the availability of mAbs also provides possibilities to trace antigen-specific B cell lineages in longitudinal samples from given individuals to evaluate immune response dynamics (2, 4). A critical step in all these analyses is the assignment of the antibody sequences to a database of germline V, D, and J genes and alleles to define their gene usage. Furthermore, since most class-switched antibodies are subject to somatic hypermutation (SHM), a central question in many studies is to what extent Ab sequences are modified by SHM, and what role this plays for antibody binding and function.

The number of IGHV genes present in rhesus macaques is currently not fully defined, despite useful databases generated by several research groups over the past years (6, 9–16). In the best-defined immunoglobulin gene region to date, the human IGH locus, the presence of pseudogenes interspersed between functional V genes, as well as frequent duplications and deletions that involve multiple IGHV genes, are known to complicate efforts to assemble genomic sequences spanning this region (17). At present, three full genomic assemblies are available for the rhesus macaque IGH locus (18–20), and these differ between each other at both gene and allele level. The challenges inherent to sequencing the IGH genomic region have led to the development of alternative approaches for immunoglobulin germline allele identification using computational inference tools such as IgDiscover, which uses data from expressed IgM libraries to identify known and novel IGHV alleles in different species (10). Inference methods are especially useful to capture allelic diversity in outbred populations, such as humans and macaques (10, 21, 22). A further advantage with inference approaches is that only functional alleles are identified since the analysis is performed on expressed VDJ repertoire data and will therefore not include unrecombined pseudogenic sequences from the IGH or other genomic regions.

Using germline gene inference from bulk Rep-seq data, we previously analyzed the IGH VDJ germline allele content in 27 rhesus macaques and 18 cynomolgus macaques and found significant variation between animals within each species (16). Of the IGHV alleles we identified in rhesus macaques, 702 of 761 (92%) were novel. Additionally, KIMDB contains 615 alleles from cynomolgus macaques. The database, the Karolinska Institutet Macaque Database (KIMDB), is accessible at: http://kimdb.gkhlab.se/ (16).

In an alternative approach, the International ImmunoGenetics Consortium (IMGT) (23) used genomic assembly information to generate a rhesus macaque IG germline gene database for which an updated version was released on July 27, 2021. The approach taken by IMGT was to identify putative IGH genes from genomic data, Mmul10/rheMac10 (20). The IMGT genes were labelled as functional (F), pseudogenic (P), or non-functional open reading frame (ORF) by genomic sequence analysis and annotated according to the estimated position on the chromosome. The Mul10/rheMac10 derived genes were supplemented with an additional 8 genes identical to genes described in the Sundling et al. rhesus macaque database (6), comprising IGHV sequences from the Gibbs et al. rhesus macaque Mmul_051212 assembly (24), which contains an incomplete IGH locus. Two additional IGHV genes, each a single nucleotide different from genes described in Sundling et al., were included, again derived from the Mmul_051212 assembly. The same animal was re-sequenced at higher coverage to produce the Mmul8 assembly (25).

Here, we analyzed the KIMDB and IMGT rhesus macaque IGHV databases for overlapping allele and gene content to identify sequence gaps that may deleteriously affect germline gene assignment and SHM determination. We examined available genomic and inferred haplotype-based evidence to identify structural variation that may affect gene content between individual macaques. Finally, we examined how gene databases constructed using either the single genomic assembly model or the multiple animal model influenced the analyses of SHM using an IgG library from a test animal that had not been used to generate either of the two databases.

## Methods

### Source of Library Data

Rhesus macaque antibody libraries analyzed in this study were deposited in the European nucleotide archive (ENA), including IgM paired-end reads (ERR2856338) and IgG paired-end reads (ERR2856339) from animal D20 (4). Databases for the assignments were downloaded from KIMDB release 1.0 and from the IMGT/VQuest release 202130-2 (27 July 2021). Sequences were converted from IMGT’s gapped alignments to ungapped germline allele sequences as well as sequence identifiers were cleaned up to contain only V gene names with the use of IgBLAST’s (v1.15.0) edit_imgt Perl script. Sanger-validated sequences were obtained from Vazquez Bernat et al. (16). Genomic assemblies for *Macaca mulatta* (Rhesus monkey) were downloaded from NCBI with identifiers GCA_003339765.3 (rheMac10), GCA_008058575.1 (RheMacS), as well as GCA_005453305.1 and GCA_005453525.2 (ASM545330v1 and ASM545352v2) representing a single animal. Therefore, genes present on either of latter two assemblies are treated in this analysis as one genome, denoted Cirelli et al., without a version number. Only the chromosome 7 sequence was extracted as this contains the macaque IGH locus, which was used for exact searches of sequences in both orientations, except for Cirelli et al. (18), for which all contigs containing V genes were searched with both forward and reverse complemented database sequences.

### Computational Analysis: Preprocessing and Genotyping

The preprocessing and germline inference from the D20 IgM and IgG libraries were performed with IgDiscover (v0.12.4) using default settings, except for 3’ end barcode length set to 21 nucleotides (nts), species set to rhesus_monkey and number of iterations set to one to produce an individualized IGHV database from the IgM library. After obtaining the germline IGHV genotype, three IgDiscover assignment runs were performed on the D20 IgG test library, using either the rhesus macaque KIMDB, rhesus macaque IMGT or the D20 individualized genotype with the same settings as previously described. The filtered tab from these runs were used to compare mutation levels obtained when assigning to the different databases.

### Database Comparisons

Databases were compared in two ways. The first approach aimed to identify identical sequences in the two databases. This included sequences that occurred within the string of longer sequences, accounting for the identification of potential truncated matches. The results were illustrated in a Venn diagram using ggVenDiagram (26) (v1.1.4) in R (27).

The second approach aimed to compare gene content of the two databases and involved complete alignments. Here, we considered exact matching to be insufficient since different alleles of a given gene could be present in the two databases. For each IGHV sequence in KIMDB, we then identified the most similar IMGT sequence by computing all vs all pairwise alignments, picking the best scoring alignment and computing the number of differences between two aligned sequences. Since the comparison concerned sequences that are highly similar, we aligned their entire length using a global alignment method. All vs all alignments were computed using BioPython’s (28) (v1.76) PairwiseAligner function with gap opening score set to -0.5, gap extension of -0.1, while end gap scores for both target and query sequence were set to zero. For each pair of genes, the best scoring alignment was selected and stored (**Supplementary Table 2**). 113 KIMDB genes, including 58 that had indefinite positional genomic locations within the macaque IGH locus, termed ‘Not Located’ (NL), were compared to 111 IMGT genes, of which 86 are denoted as functional. For each gene in KIMDB or IMGT the best scoring alignments and distances were reported (**Supplementary Table 2**). Tables were used as input data for custom plotting scripts in R with ggplot (v3.3.5) (29). IMGT genes that were distant from any sequence found in KIMDB were also used as queries to find the corresponding best matches from KIMDB. To obtain an estimate of the median distance for alleles of a given gene, we calculated the Levenshtein distance and denoted this set as the “within gene” set. We also computed the distance between all allele pairs of different genes of the same family as the “between gene” set. The median distance ‘within gene’ was close to 5nt for all IGHV families. We used this distance as a criterion to group sequences that were likely to be allelic variants of the same gene to compare gene content in the IMGT and KIMDB databases and the presence of genes in the reference assemblies.

### Generation of an Individualized IGHV Genotype From the Test Animal

To define the IGHV genotype and generate an individualized database for animal D20, an IgM library was generated as described (4, 26) and analyzed with IgDiscover using the rhesus macaque KIMDB as the input database. A total of 91 IGHV alleles were identified. To build a tree of the IGHV alleles from D20, the 91 alleles were aligned with the iterative multiple sequence alignment tool MAFFT (v7.487) (27) FastTree (v2.1.11) (28) was used with default parameters; starting with a neighbor-joining algorithm and improving topology and branches with a maximum-likelihood approach. To visualize the tree, the ggtree (v3.13) (29) package for R was used. Gene families were marked with different colors and alleles that overlapped with known IMGT alleles were colored red.

### SHM Levels Comparisons

For the comparison of SHM levels, we used an IgG library from rhesus macaque D20, an animal that had not been used to generate the KIMDB database or the IMGT database. Three analyses were performed with the D20 IgG library. First, the library was assigned to the individualized D20 database to identify the appropriate germline assignment for each sequence read. For each assigned gene from D20 run, read identifiers were extracted and used to obtain SHM related values from the D20 IgG runs with KIMDB and IMGT as starting databases. All sequences assigned to a germline sequence in the initial individualized run were grouped into allele specific bins. The same allele specific binned sequences could then be traced in the subsequent KIMDB and IMGT assigned analyses, thereby enabling the SHM per gene to be determined based on the V_errors column of the filtered table output. Genes and their median V mutations, determined as number of differences reported by IgBLAST were compared to the closest IGHV allele in the database.

### Inferred Haplotype Analysis

Inferred haplotype analysis of the D20 IgM library was performed using the IgDiscover plotallele module as described (16) using IGHJ5-4*02 and IGHJ5-4*03 as chromosomal anchors. The IGHV gene order is an estimation, based on a composite gene order from the three published macaque assemblies.

### Validation Using Genomic Assemblies

RheMac10 (20) and rheMacS (19) genomic assemblies were downloaded and pre-processed into FASTA files. Sequences from chromosome 7 for rheMac10 and rheMacS were used as a database for BLASTn+ 2.12.0, to query KIMDB sequences. For the Cirelli et al. assembly (18), both primary and alternate assemblies were used with all contigs, because of lack of defined chromosomes. Results were saved in tabular format and processed with a custom script to compute a distance between query and the matching genomic sequence. Due to the local nature of BLAST, distance is defined as the sum of not-aligned residues from a query and the number of aligned residues that differ from a genomic sequence; including gaps which are counted as a difference. To remove spurious matches all the sequences were filtered such that no more than 5 nt differences were allowed.

## Results

### Comparison of the IMGT and KIMDB Databases for IGHV Allele and Gene Content

**Figure 1:**
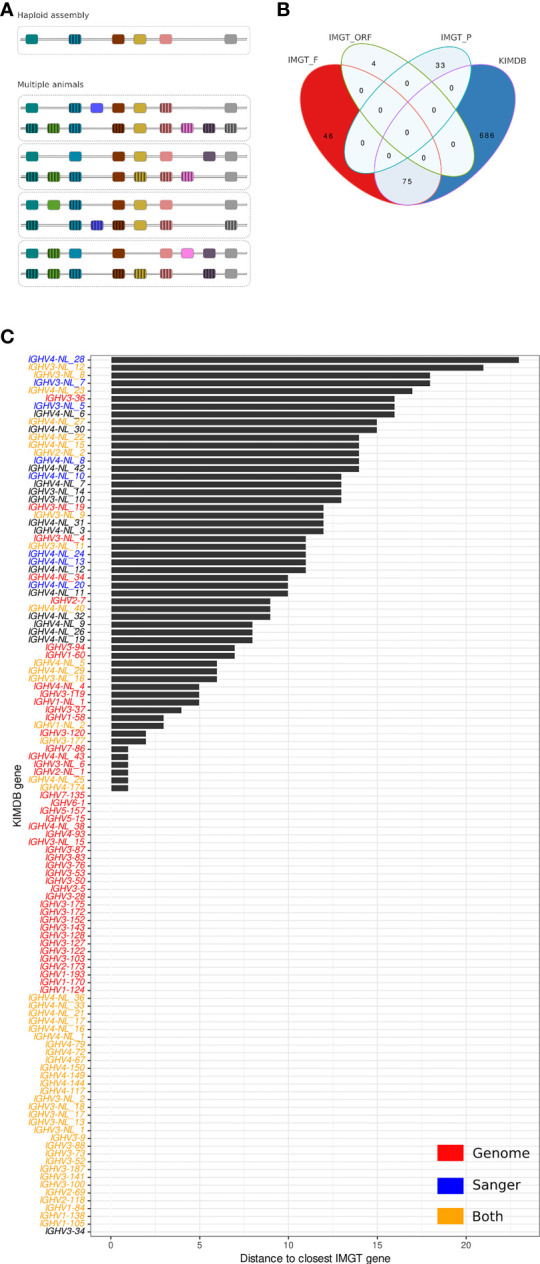
Comparison of the rhesus macaque IMGT database and KIMDB. **(A)** Schematic of reference database approaches. Database construction following expression-based germline inference from multiple animals, where individual structural variations are cross-compensated by other animals compared to database construction following identification of all genes present in a haploid IGH assembly that lacks genes due to structural variation. **(B)** Venn diagram showing exact matches between IMGT/VQuest alleles marked as functional (IMGT_F), open reading frame (IMGT_ORF) or pseudogenic (IMGT_P) and alleles in the rhesus macaque KIMDB. **(C)** Distance in nucleotide sequence between closest matching IGHV alleles of two genes from the rhesus macaque IMGT database and KIMDB. Genes that have at least one allele less than 6 nucleotide differences across the entire query sequence were assumed present in public genome assemblies and are labeled in red, IGHV sequences that were Sanger sequence-validated in Vazquez Bernat et al. are labeled in blue, and IGHV sequences that were both present in public genome assemblies and Sanger-validated are labeled in orange. The remaining genes are labeled in black.

Structural and copy number gene variation in the IGH locus has been demonstrated previously in both human and macaques (16, 17). This factor has specific consequences in the construction of a reference database suitable for high quality IG gene assignments. Even a perfectly assembled haploid IGHV locus is limited to the genes present on that locus. If structural variation is common, then any genes missing in one assembly will be absent from a reference gene database based on that assembly. In contrast, the combination of individualized germline genotypes from multiple individuals will ensure the presence in the database of allelic representatives of genes that may be frequently missing in individual animals due to common structural variations (**Figure 1A**), in addition to ensuring a high level of allelic variation in the database.

The first step in antibody VDJ sequence analysis involves assigning sequences to a germline gene database to define gene and allele usage of each antibody. Here, we compared two immunoglobulin IGHV germline gene databases for rhesus macaque antibody analysis, IMGT (23) and KIMDB (16). For the former, we used the rhesus macaque IMGT/VQuest database, released on July 27, 2021, which comprised 111 genes and 158 IGHV alleles, of which 121 alleles and 86 genes were denoted functional (F), 3 genes and 4 alleles were denoted as open reading frames (ORF), and 26 genes and 33 alleles were denoted as pseudogenes (P). The KIMDB, which was produced by germline gene inference using the IgDiscover software and several subsequent validation steps, comprised 761 alleles and 113 putative genes, all defined as functional. We defined functional alleles as full-length IGHV sequences without stop codons that were present in multiple independent VDJ recombination events in expressed IgM libraries, since this confirmed that the alleles located to the IGH locus and contained functional recombination signal sequences (16).

The two databases were first compared for exact allelic matches, accounting for the potential presence of sequences that were not full-length in one of the databases. As shown in the Venn diagram in **Figure 1B**, 75 F-marked IMGT alleles were present in KIMDB, while none of the IGHV ORF or P denoted alleles were found in KIMDB. Furthermore, 46 F-marked IGHV alleles in IMGT were not present in KIMDB, while 686 IGHV alleles were present in KIMDB but missing from the IMGT database (**Figure 1B**). The 75 IGHV alleles that were identical between IMGT and KIMDB have different names due to the technical approaches used to create the corresponding databases and the positional nomenclature used to name the rheMac10 derived candidate genes. The identical alleles are listed in **Supplementary Table 1**. Analysis of inter and intra-gene variation in the KIMDB shows that the median difference between alleles of the same gene is around 5 nts for all five gene families, while the median difference between genes in the same IGHV gene family is 42 nt for IGHV1, 21 nt for IGHV2, 49 nt for IGHV3, 21 nt for IGHV4 and 14 nt for IGHV5 (**Supplementary Figure 1**).

We next grouped the alleles according to genes and compared the two databases for best matching gene content. The result for each KIMDB gene is shown as distance to the closest IMGT gene by comparing the best-matching alleles from each gene (**Figure 1C**). We found that 56 of 113 genes in KIMDB had an allelic variant that was identical to an allele in IMGT, shown in the graph as zero distance. This included several genes denoted as NLs in KIMDB. The NLs were defined in our previous study based on clusters of alleles that were markedly different from genes present in the starting database (16). These may represent unique genes that were present in the 27 animals used to create the KIMDB, but which were absent in the ASM545330v1 and ASM545352v2 assemblies (18). In our previous study, we confirmed several NLs by targeted PCR, cloning and Sanger sequencing (**Figure 1C**). Here, we extend this analysis and examined gene content variation between the three published macaque genomic assemblies. Specifically, the Cirelli et al., rheMac10 and rheMacS assemblies contained 53, 43 and 45 of the genomically located IGHV genes, respectively, and 14, 18 and 28 of the NLs (**Supplementary Figure 2**). A group of 11 genes marked in gray bars differed by less than 5 nucleotides from the closest IMGT gene (**Figure 1C**). This is within the distance typically observed for allelic variation (**Supplementary Figure 1**). Another set of genes, shown in black bars, differed between 5 and 25 nucleotides from the closest IMGT gene (**Figure 1C**). This genetically distant set of KIMDB genes included genes from the rhesus macaque IGH assembly published by Cirelli et al. (18). These included IGHV2-7, IGHV3-36, and IGHV3-94. The complete data set used to generate **Figure 1C** are described in **Supplementary Table 2**.

We also performed the analysis in a reciprocal manner, asking if there were any functional IMGT genes that could not be matched with genes in KIMDB. We identified 5 F-marked IMGT genes that that did not have a closely related sequence in KIMDB: IGHV1-69, IGHV3-12, IGHV3-35, IGHV3-108 and IGHV3S40 (**Supplementary Figure 3** and **Supplementary Table 2**). These genes displayed distances between 10 and 40 nucleotides to the closest sequence in the KIMDB, demonstrating that none of the alleles in KIMDB were closely related to these five IMGT genes. However, a BLAST search for these five IGHV sequences found zero matches (above 90% identity) to mRNA derived sequences from recombined antibodies from any published study of expressed macaque IGHV sequences at the time of writing, raising a question regarding the functionality of these five IMGT rhesus macaque genes. It is important to note that there is evidence for the presence of these sequences at the genomic level in several rhesus assemblies. All five IMGT genes are present in the rheMac10 assembly. IGHV3-12 and IGHV3S40 are also present in both ASM545330 and rheMacS, while IGHV1-69 and IGHV3-35 are present in ASM545330 but not in rheMacS. The presence of these genes at the genomic level combined with the absence of the same sequences in expressed libraries suggests the possibility that they may be non-functional or, alternatively, utilized very rarely.

### IGHV Germline Alleles and Analysis of IgG Repertoires in a Test Rhesus Macaque

**Figure 2:**
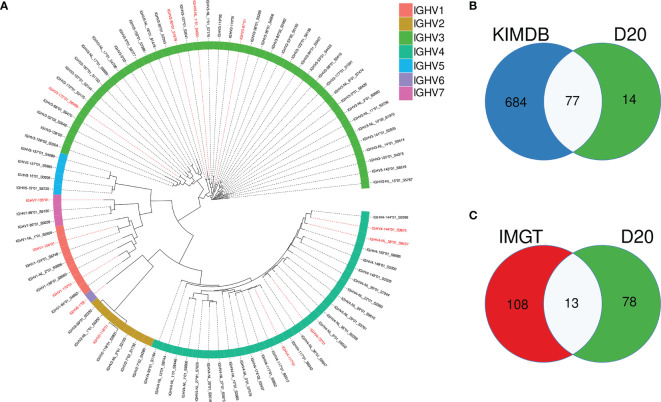
Overlap of IGHV alleles in animal D20 with alleles present in the rhesus macaque IMGT database and KIMDB. **(A)** An approximate maximum likelihood tree of D20 IGHV alleles with alleles that are identical to IMGT alleles labeled in red. **(B)** Venn diagram showing the overlap between IGHV alleles present in D20, marked in green, and KIMDB, marked in blue **(C)** Venn diagram showing the overlap between IGHV alleles present in D20, marked in green, and the IMGT rhesus macaque database marked in red.

To model the impact of germline databases for VDJ allele assignments and analysis of SHM, we selected one rhesus macaque, D20, which was not part of the 27 animals used to generate KIMDB. We defined the germline IGHV allele content in D20 using the IgDiscover software (v0.12.4) and identified 91 IGHV alleles, representing 66 IGHV genes. The full allelic content of D20 is shown in a phylogenetic tree (**Figure 2A** **)**. Of the 91 alleles, 77 were identical to alleles already present in KIMDB and 14 alleles were novel (**Figure 2B**). Of the 91 D20 alleles, only 13 were present in the IMGT rhesus macaque database (**Figure 2C**). Of these 13 alleles, marked in red in the phylogenetic tree, two were NL alleles, IGHV3-NL-1*01_S4331 and IGHV4-NL-38*01_S8437.

Using a germline gene database that lacks IGHV alleles can result in incorrect clonal assignments and an overestimation of SHM levels as previously discussed (30). However, it is potentially far more problematic if the database lacks IGHV genes expressed in the animal as allelic absence in the reference database will result in expressed sequences being assigned to alternative alleles of the same gene. Absence of a gene, however, means there are no alternative alleles of that gene available for assignment and the sequence will instead be erroneously assigned to a different gene that is the closest in sequence identity to the expressed sequence. In both these situations the assignment will result in an incorrect estimation of SHM, however the difference between alleles of the same gene is far less than the difference between genes. (**Supplementary Figure 1**).

**Figure 3:**
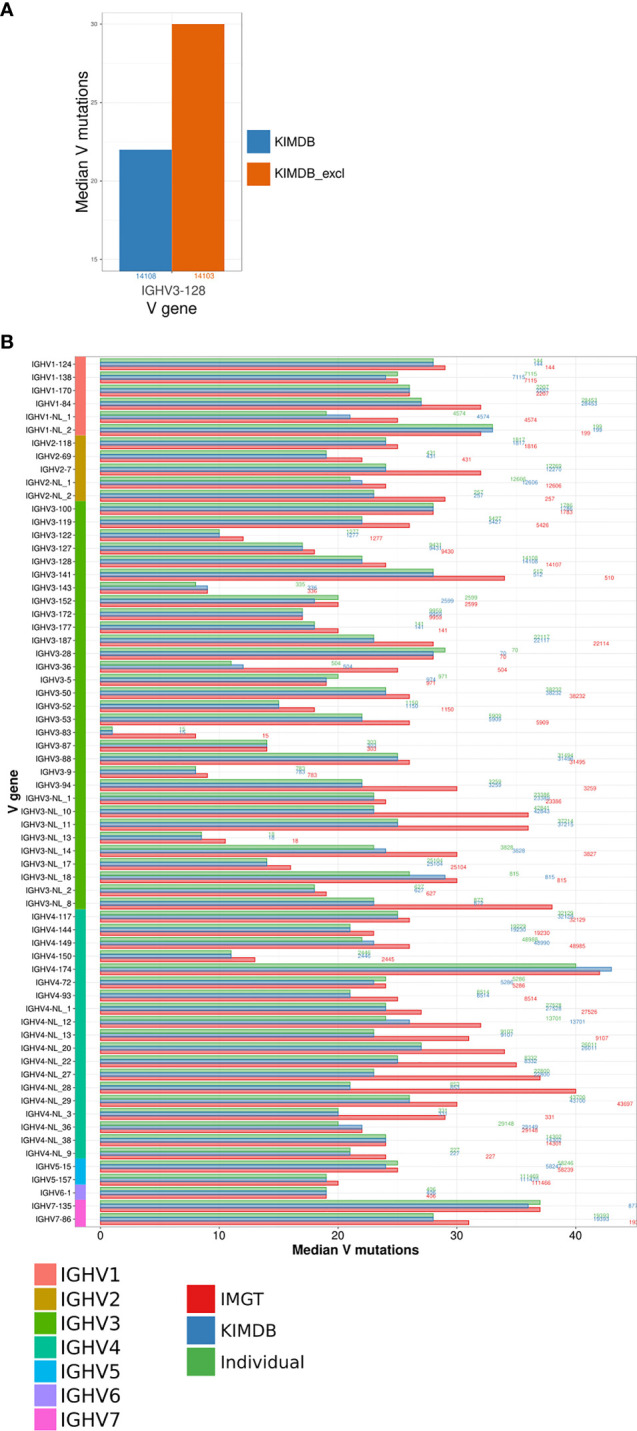
Evaluation of the number of mutations from germline using different databases. **(A)** Comparison of differences in the estimation of SHM using the full KIMDB, marked in blue and KIMDB_excl, a version of KIMDB that has all alleles of the gene IGHV3-128 removed, marked in orange. **(B)** IgG sequences from the D20 animal were assigned to the animal’s individual IGHV database, shown in green, KIMDB, shown in blue, or the IMGT rhesus macaque database, shown in red. The average number of mutations in the IGHV sequence after assignment to each of the databases is shown by the median V mutations count. The numbers to the right of each set of gene specific bars indicate the number of reads for that gene in the IgG library.

To model the impact of missing genes, we first generated a version of the rhesus macaque KIMDB where we purposefully removed one IGHV gene, IGHV3-128, for which we had high sequence counts in the D20 IgG library. We assigned all IgG sequences to KIMDB and KIMDB lacking IGHV3-128 (termed KIMDB-3-128). We then extracted all 14108 IgG sequences that were assigned to IGHV3-128 in KIMDB and compared their mutation level to the levels obtained when assigning the same sequences to KIMDB-3-128. The analysis illustrated clearly that the median number of mutations in the IgG sequences was higher (median of 30 instead of 22 mutations) if the database was missing this gene, since 99% of those reads instead get assigned to the next closest gene, IGHV3-88 (**Figure 3A**).

To further assess the impact of potentially missing genes, we assigned the full D20 IgG library to three databases, IMGT, KIMDB and the D20 individualized database, and examined the median number of mutations in IgG sequences representing all genes. The results show a markedly higher median number of mutations for several genes when using the IMGT database compared to KIMDB or to the animal’s own genotype of alleles. This analysis illustrated that the use of a database that lacks genes results in greatly overestimated mutation levels. Many of the genes that showed high mutation levels belonged to the IGHV3 and IGHV4 families, suggesting that these gene families are more incomplete in IMGT. For example, sequences from the genes IGHV3-NL_8 and IGHV4-NL_28 resulted in median V mutations 15 and 19 nucleotides higher, respectively, for the IMGT database assignment compared to the individualized or KIMDB database assignment (**Figure 3B**). For most genes, assignments to the KIMDB and the D20 individualized database resulted in similar levels of mutations, supporting that KIMDB is more complete in its IGHV gene content even though it still likely lacks alleles present in additional macaques.

**Figure 4:**
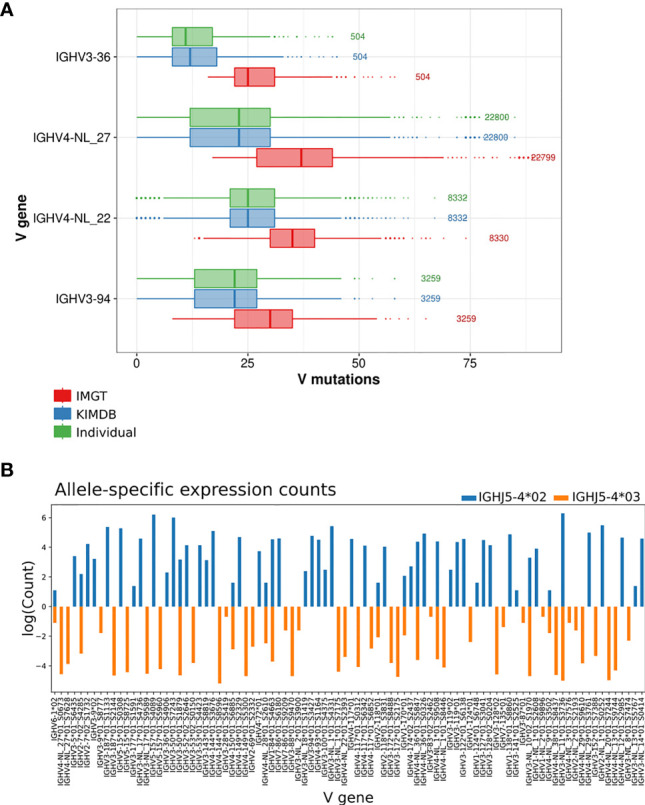
Evaluation of V mutations from germline sequence using different germline gene databases and inferred haplotyping. **(A)** The average number of mutations in antibody sequences using four IGHV genes that are absent in the IMGT database after assignment to the animal’s individual IGHV database, KIMDB or the IMGT rhesus macaque database. The numbers to the right of each gene indicate the number of reads for that gene in the IgG library. **(B)** Inferred haplotype analysis of macaque D20. Illustration of inferred haplotyped allele content based on germline V allele VDJ recombination counts with heterozygous J alleles, IGHJ5-4*02 (in blue) or IGHJ5-4*03 (orange).

To further illustrate this, we selected 4 IGHV genes that were frequently used in the repertoire and where the sequence differences between IMGT and KIMDB were among the largest, IGHV3-36, IGHV4-NL_27, IGHV4-NL_22, and IGHV3-94, and found an overestimation of median V mutations by 14 (median difference of 4.7% SHM), 14 (4.7% SHM) and 10 (3.3% SHM), 8 (2.7% SHM) nucleotides respectively when the IMGT database is used in contrast the individualized D20 database. The results demonstrate that the level of SHM of many genes expressed in D20 would be misinterpreted as having SHM levels overestimated by approximately 5% when an incomplete database is used (**Figure 4A** **)**.

To illustrate the issue of structural variation at the IGHV locus, we performed inferred haplotype analysis (16, 31, 32) of the D20 IgM library. The animal was heterozygous for the IGHJ gene IGHJ5-4, and we were able to utilize this to chromosomally separate the IGHV alleles that were located on either the IGHJ5-4*02 or the IGHJ5-4*03 containing chromosomes. 7 genes, IGHV2-NL_2, IGHV3-36, IGHV3-NL_8, IGHV4-NL_3, IGHV4-NL_12, IGHV4-NL_13 and IGHV4-NL_20, were present hemizygously in D20, but were absent from the rheMac10 assembly (**Figure 4B** and **Supplementary Figure 2**). One gene, IGHV4-NL_28, was present homozygously in D20, but absent in rheMac10, and one gene, IGHV2-7 was heterozygous in D20 but absent in rheMac10.

The data shown here, indicating significant gene differences between the two chromosomal IGHV loci within a single animal, are consistent with the level of differences of IGHV gene content found between different macaque assemblies (**Supplementary Figure 2**). All the previously mentioned genes that were present in D20 but absent in rheMac10 demonstrated large increases in median V mutation level when assigned using the IMGT database compared to either the D20 individualized database or the KIMDB (**Figure 3B**).

## Discussion

Deep sequencing of adaptive immune receptor repertoires allows computational inference of IGHV alleles directly from expressed VDJ arrangements using tools such as IgDiscover (10), TiGger (33) and partis (22). Inference approaches are amenable to the analysis of larger population groups, which is necessary to fully understand IG genetic variation in outbred species such as humans and macaques (10, 16, 21, 34–36). We previously generated the KIMDB macaque database, by applying IgDiscover germline inference analysis to 27 rhesus macaques, from which we generated two independent IgM libraries for each animal. This analysis confirmed the high allelic diversity in the macaque IGH locus that we previously identified (10). Additionally, inferred haplotype analysis performed in the recent study revealed the frequent presence of IGHV structural diversity in macaques (16). In a recent study, we also identified expansion of the IGHV4 family in rhesus macaques and described gene usage frequencies in the naïve B cell repertoire (37).

Structural and copy number variation inevitably provides an obstacle to the creation of a comprehensive gene map of a locus. If structural variation is frequent and diverse in terms of which genes it affects, it may be difficult or even impossible to identify all IGHV genes in one animal. This becomes even more difficult if a restricted set of genomic assemblies are used to identify all the V genes present in such diverse species. In the current study, we show that the gene diversity revealed by inference studies is reflected in the gene content of available macaque reference genomic assemblies (18–20). A total of 90 unique macaque genes (including both genomically located genes and NLs) from the KIMDB were identified present in at least one of the macaque genomic assemblies analyzed, Cirelli et al., rheMac10 and rheMacS. However, no single reference assembly contained all 90 genes, with the assemblies lacking 23, 29 and 17 of these genes respectively (**Supplementary Figure 2**).

The variation in gene content between macaque assemblies reflects the structural variation identified by Vazquez Bernat et al. (16). In that study structural diversity that impacted gene content was illustrated using inferred haplotype analysis in two animals. One animal Rh4 showed hemizygous presence of 3 genes, IGHV2-7, IGHV3-36 and IGHV2-118, that were absent from the rheMac10 assembly. A separate animal, D07 also showed hemizygous presence of IGHV2-7 and IGHV3-36, as well as two additional genes, IGHV1-105 and IGHV4-93 that also are absent from rheMac10.

In the current study, we extended this haplotype analysis to include an additional animal, D20 that showed the presence of 9 genes that were absent in the rheMac10 but found in one of the other two assemblies (IGHV2-118, IGHV2-7, IGHV2-NL_2, IGHV3-36, IGHV3-NL_8, IGHV4-NL_22, IGHV4-NL_27, IGHV4-NL_29, IGHV4-NL_38 (**Figure 4B**). The inferred haplotype identification of the same genes in a hemizygous state in multiple animals but absent in others is consistent with the presence of common structural variants in the macaques. However, the variety of hemizygous gene presence in different animals is indicative of multiple independent structural variants in the species. It is important to note that both IGHV gene content and gene order are highly discrepant between the three current reference assemblies produced using PacBio sequencing approaches. The lack of consensus for the order of IGHV genes suggests that many additional assemblies using a variety of animals with different gene content are required to arrive at a conclusive gene order in the locus. Even then, we may have to contend with structural differences between animals that disrupt the order.

The presence of structural diversity that impacts individual gene content has important consequences for the construction of reference databases. Critically, the consequences for VDJ gene assignment accuracy using a gene restricted database derived from a single or low number of animals compared to one derived from multiple animals has, to date, not been quantified in detail. We identified allelic sequences corresponding to genes absent from the Cirelli et al. database by performing phylogenetic analysis of all IGHV alleles identified in the 27 rhesus macaques combined with an additional set of alleles described in the earlier study (10). This revealed clusters of alleles that were distinct from allelic clusters of known genes. We selected a representative sequence from each distinct cluster, NLs, representing genes where no genomic location is yet identifiable within the Cirelli et al. assembly. If the germline NL sequences that are absent from the assembly are associated with common structural variants, we should expect to find them in genomic assemblies from different macaques. Indeed, when we searched three currently available rhesus macaque genome assemblies (18–20) for the 58 NLs using the previously defined criterion of up to 5 nt distance from any alleles in KIMDB, we found 36 of the NLs in at least one of the assemblies. A full list of which genes, including the NLs, are present in which genomic assemblies is described in **Supplementary Table 2**. 56 genes (genomically located and NLs) were found in both the IMGT database and KIMDB with 100% sequence match to an allelic variant. However, several genes in KIMDB were missing from the IMGT database, as shown by a high sequence distance when searching for the closest KIMDB gene. This includes several of the genes described in the Cirelli et al. assembly, including IGHV3-36, IGHV2-7, IGHV3-94.

Structural variation at the IG locus also represents a challenge for IGHV gene nomenclature. The positional approach for naming genes, utilized by IMGT, poses problems when additional haplotypes, containing genes that are missing from previous analyses, are found or when alternative assemblies of the same reference animal are created that reveal the original gene order is incorrect. An alternative strategy is to name newly identified genes in the order they were discovered. For genomic approaches, there is also a risk that mistakes are made when annotating genes as functional or non-functional as the analysis is not coupled to independent analyses of expressed Ab repertoires. In the current list of IMGT rhesus macaque IGHV genes, we identified 5 genes that are denoted as functional that did not have a counterpart in KIMDB in terms of sequence similarity. However, following BLAST search analysis, we found no record that these genes are utilized in recombined VDJ sequences.

We also compared the rhesus macaque KIMDB with the IMGT/VQuest rhesus macaque database released on July 27, 2021, for analyses of SHM levels in a test IgG library. We found that the absence of numerous common IGHV genes in the IMGT database greatly influenced the analysis of SHM due to mis-assignments of expressed antibody sequences that use these genes (**Figure 3B**). In previous work, we observed considerable allelic variation between individual macaques with several novel alleles discovered in every additional animal analyzed. It was therefore unsurprising to identify 14 novel IGHV alleles in D20, the test animal used in this study, that were not present in KIMDB. However, all 14 alleles were close variants 1-3 nucleotides away from genes already defined in KIMDB, suggesting that KIMDB has a high if not full coverage of IGHV genes, albeit not of all alleles of these genes. The creation of an individualized IGHV germline allele genotype for D20 allowed us to address SHM in an IgG library generated from this animal. Assignment to the animal’s own germline allele database provides the “gold standard” for SHM calculation as this enables all IgG sequences to be assigned to the correct germline allele. This is particularly important for lineage-based vaccine design where precise information about germline-encoded residues, IGH VDJ allele usage, B cell clonal relationships and SHM are required. However, since KIMDB contains representative alleles of all currently known expressed genes, using this database without performing individualized genotyping should result in minimal error rate (1-2%) of the assigned sequences.

When comparing assignments to the individualized database, KIMDB and the IMGT rhesus macaque database the results were clear. Very similar levels of SHM were obtained when using the individualized IGHV germline allele database and KIMDB. For germline genes that were present in D20, KIMDB, and IMGT we saw identical levels of SHM. However, assignments to IMGT resulted in notably higher SMH levels for those genes that were present in D20 and KIMDB but absent from the IMGT database (**Figure 3B**) resulting in an overestimation of SHM by approximately 5% for several genes. This result is certainly an improvement compared to an earlier iteration of the IMGT macaque database (38); however, the continued absence of numerous common macaque genes should be addressed to ensure accuracy of SHM calculation and lineage tracing.

The diversity of IGHV gene content between the different macaque assemblies shown in this report is entirely consistent with the level of variation revealed by germline inferral approaches using individual animals and within single animals using inferred haplotype analysis (10, 16). The issue of a comprehensive gene nomenclature in macaques is a currently unresolved question; however, our results show that a combination of Rep-Seq based inference to identify functional IG sequences, and genomic based assembly analysis to identify unique genes, offer a viable approach to achieve the future goal of producing a comprehensive rhesus macaque germline reference database with an agreed genomic based nomenclature.

Genetic diversity within the adaptive immune receptor genes of outbred animal species is a hitherto understudied topic. The utility of specific genes in counteracting constantly evolving pathogens is important as high levels of individual diversity can enable species survival during disease epidemics. Inbred animal models, frequently utilized in immunological research, such as the C57BL/6 or BALB/c laboratory strains of mice should, by design, not show intra-individual diversity, although IGHV differences between strains is clear (10, 39, 40). The extent of adaptive immune gene diversity in outbred species is only recently being quantified (10, 16, 21). The results presented here demonstrate the importance of using a large cohort of animals for construction of reference germline gene databases in outbred species and highlight how the lack of database diversity can severely impact biological conclusions.

## Data Availability Statement

Publicly available datasets were analyzed in this study. This data can be found here: https://trace.ncbi.nlm.nih.gov/Traces/sra/?run=ERR2856338 https://trace.ncbi.nlm.nih.gov/Traces/sra/?run=ERR2856339/ https://www.ncbi.nlm.nih.gov/assembly/GCF_003339765.1/ https://www.ncbi.nlm.nih.gov/assembly/GCA_008058575.1/ https://www.ncbi.nlm.nih.gov/assembly/?term=GCA_005453305.1/ https://www.ncbi.nlm.nih.gov/assembly/GCA_008058575.1/ https://www.ncbi.nlm.nih.gov/assembly/?term=GCA_005453305.1 http://kimdb.gkhlab.se/ http://www.imgt.org/download/V-QUEST/.

## Ethics Statement

Macaque blood samples were obtained from macaques housed in different animal facilities: E01-10, D7-19 in the Astrid Fagreaus Laboratory (AFL) approved by the Association for Assessment and Accreditation of Laboratory Animal Care (AAALAC), Rh1-6 at the CEA, France and A01-05 in the Oregon National Primate Research Center (OHSU), USA. Ethical permits for the work in this study are as follows: Samples from AFL/KI and Primgen: Stockholms Norra Djurförsöksetiska nämnd: N275/14 - Animals: D7, D8, D11, D15, D16 and D19, Stockholms Norra Djurförsöksetiska nämnd: N193/16 - Animals: E01-E10; Samples from CEA: APAFIS#3132-2015121014521340v2 - Animals: Rh1-Rh6; Samples from OHSU, IACUC: TR01_IP00000028 - Animals: A01-A05.

## Author Contributions

Conceptualization, MK, MC, and GKH. Methodology: MK, Analysis: MK, MC and GKH, Manuscript writing: MK, MC, and GKH. All authors contributed to the article and approved the submitted version

## Funding

This work was funded by a Distinguished Professor grant from the Swedish Research Council (2017-00968), grants from the National Institutes of Health (HIVRAD 1P01AI157299 and CHAVD UM1Al144462), and a European AIDS Vaccine Initiative (EAVI) 2020 (681137) grant.

## Conflict of Interest

The authors declare that the research was conducted in the absence of any commercial or financial relationships that could be construed as a potential conflict of interest.

## Publisher’s Note

All claims expressed in this article are solely those of the authors and do not necessarily represent those of their affiliated organizations, or those of the publisher, the editors and the reviewers. Any product that may be evaluated in this article, or claim that may be made by its manufacturer, is not guaranteed or endorsed by the publisher.

## Supplementary Material

Supplementary Figure 1 The box plot showing distributions of smallest Levensthein distance ‘between genes’ (black frames) and between alleles of the same gene ‘within gene’ (gray frames). Medians are shown with horizontal bars and the IGHV1-5 families are labelled in different colors.

Supplementary Figure 2 Presence of IGHV genes according to the defined criterion in three different rhesus macaque genome assemblies, Cirelli et al, rheMac10 and rheMacS. The column to the left show genes described in Cirelli et al, and the column to the right show NLs. Presence is shown in black and absence in gray.

Supplementary Figure 3 Five IMGT genes denoted as functional that were genetically distant from any sequence present in KIMDB and the nucleotide distance to the closest KIMDB allele.

Supplementary Table 1 Exact allele matches between KIMDB and IMGT.

Supplementary Table 2 Database comparisons and reference assembly matching.
