# Supplementary figures and images for: Addressing IGHV Gene Structural Diversity Enhances Immunoglobulin Repertoire Analysis: Lessons From Rhesus Macaque
Source: Front Immunol. 2022 Mar 28;13:818440. doi: 10.3389/fimmu.2022.818440 (PMC8995469; doi:10.3389/fimmu.2022.818440)

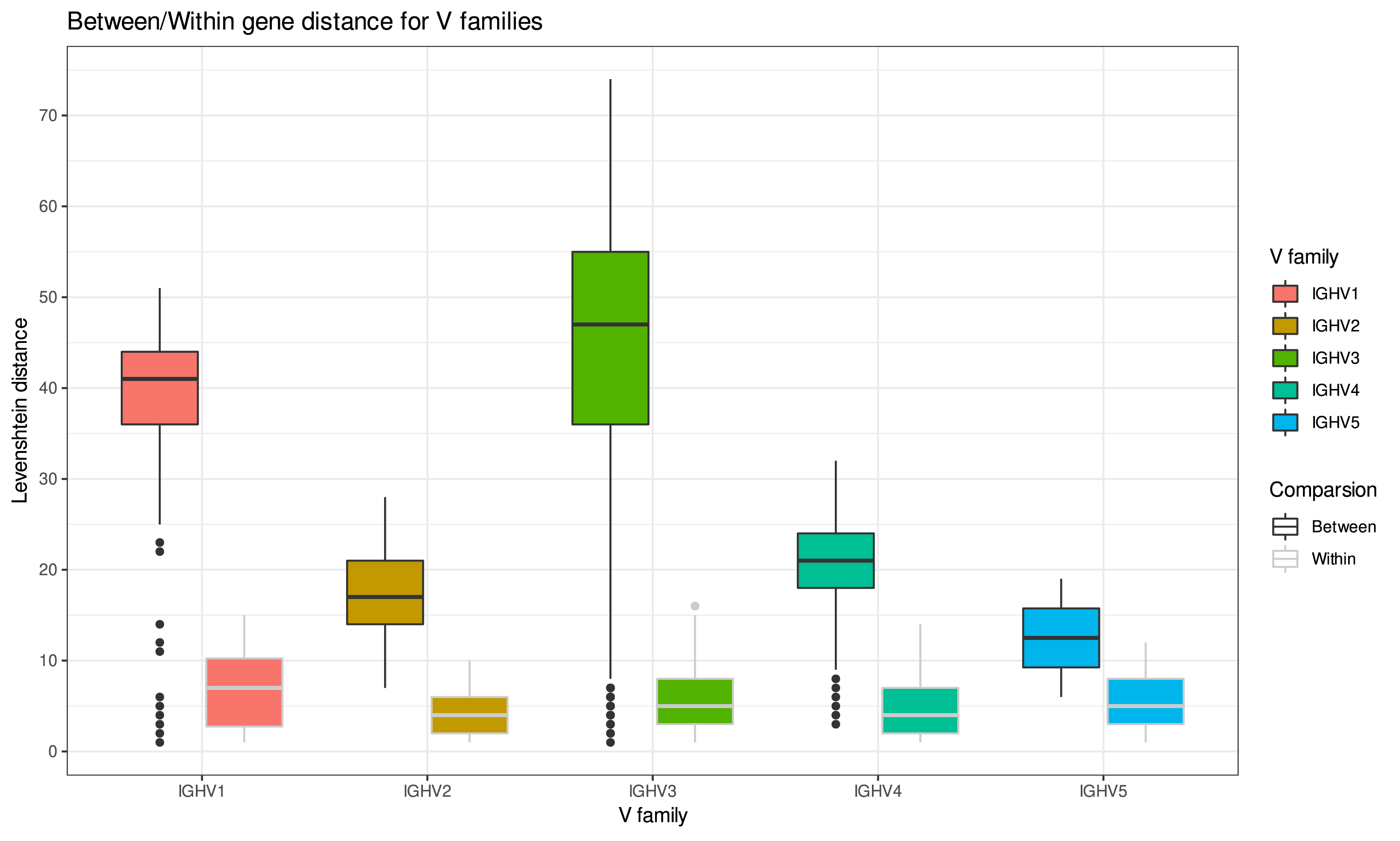

Supplement: Supplementary Figure 1 — The box plot showing distributions of smallest Levensthein distance ‘between genes’ (black frames) and between alleles of the same gene ‘within gene’ (gray frames). Medians are shown with horizontal bars and the IGHV1-5 families are labelled in different colors. [file Image_1.jpeg]

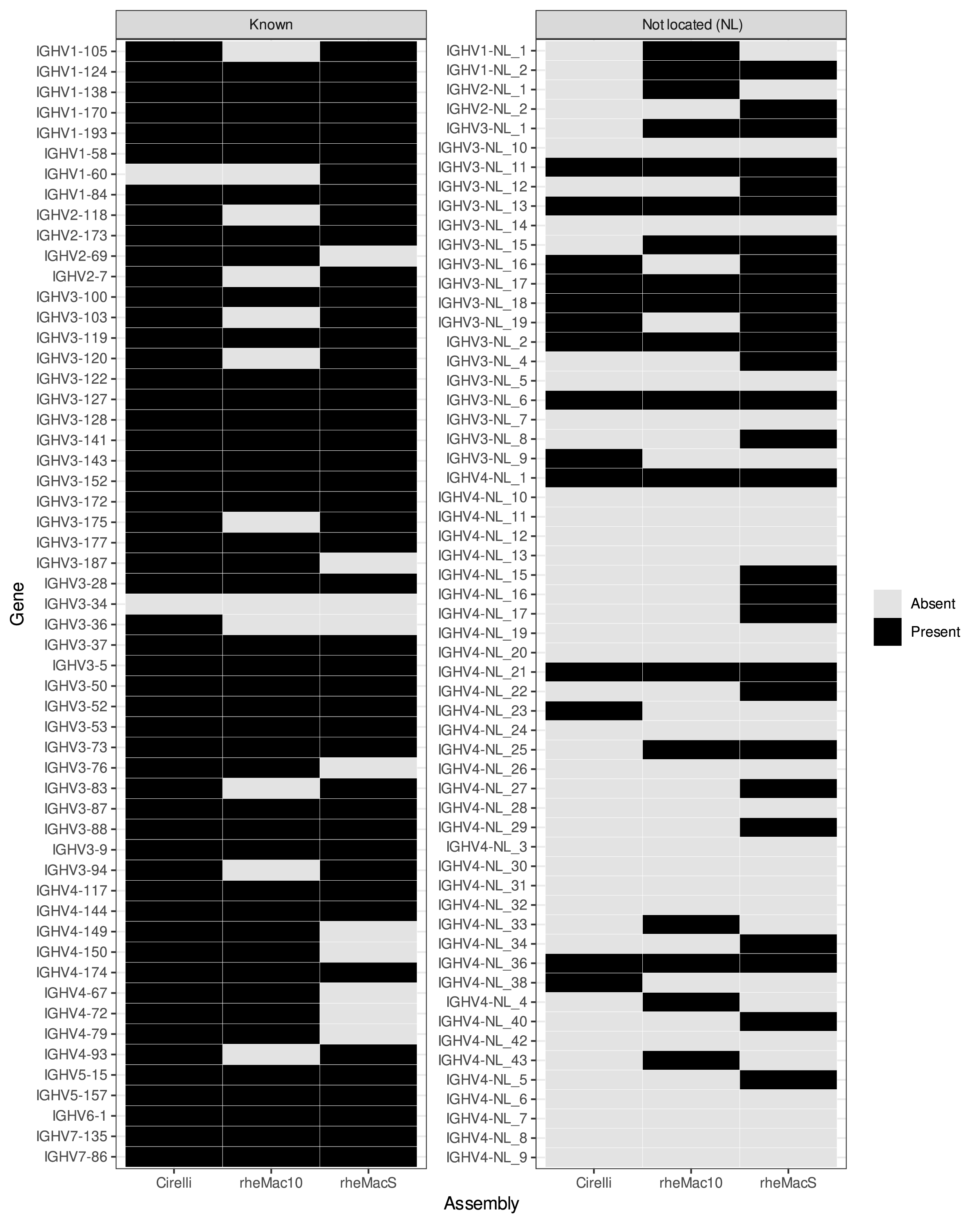

Supplement: Supplementary Figure 2 — Presence of IGHV genes according to the defined criterion in three different rhesus macaque genome assemblies, Cirelli et al, rheMac10 and rheMacS. The column to the left show genes described in Cirelli et al, and the column to the right show NLs. Presence is shown in black and absence in gray. [file Image_2.jpeg]

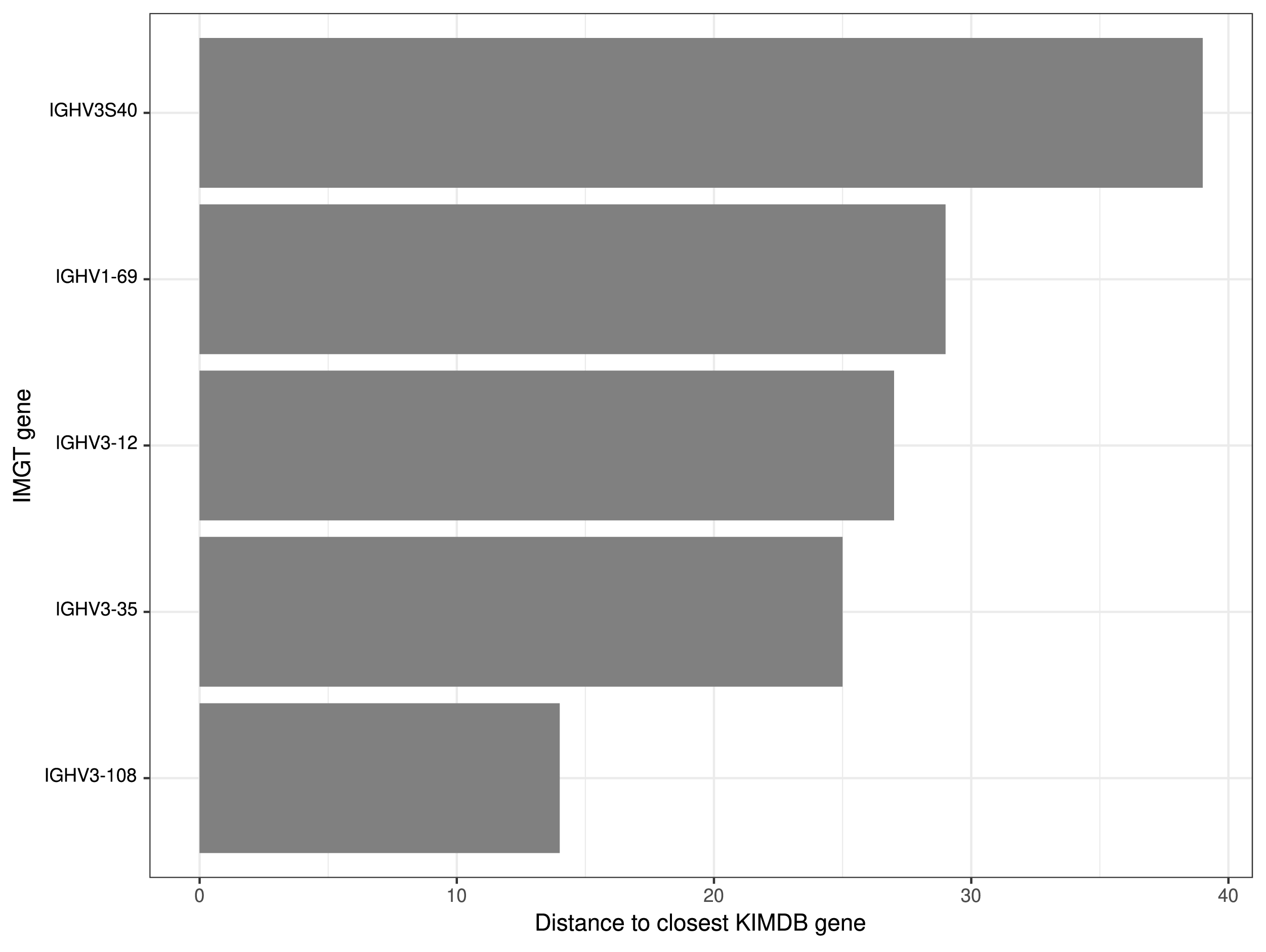

Supplement: Supplementary Figure 3 — Five IMGT genes denoted as functional that were genetically distant from any sequence present in KIMDB and the nucleotide distance to the closest KIMDB allele. [file Image_3.jpeg]
